# Potential benefit of hormonal therapy for non-uterine soft tissue sarcoma (STS) – a case report and literature review

**DOI:** 10.1186/2193-1801-2-536

**Published:** 2013-10-17

**Authors:** Li Li, Isaiah P Schuster, Robin Jacob, Kenneth H Hupart, Vladimir Gotlieb

**Affiliations:** Department of Medicine, Nassau University Medical Center, East Meadow, NY USA; School of Medicine, Stony Brook University, Stony Brook, NY USA; Division of Hematology and Oncology, Howard University Hospital, Washington, DC USA; Division of Endocrinology, Metabolism and Diabetes, Nassau University Medical Center, East Meadow, NY USA; Division of Hematology and Oncology, Nassau University Medical Center, East Meadow, NY USA

**Keywords:** Soft tissue sarcoma, LMS, Estrogen receptor, Hormonal therapy

## Abstract

The expression of hormone receptors (HR) is considered a good prognostic marker in uterine sarcoma. Hormonal therapy is widely employed in the therapy of HR positive breast and gynecologic cancers, however, there is little information concerning hormonal therapy in HR positive extrauterine sarcoma.

A 55-60 year age group female presented with an estrogen receptor positive metastatic retroperitoneal leiomyosarcoma (LMS). She was treated with four cycles of a combination of Gemcitabine and Paclitaxel. Her disease remained stable for 29 months when tamoxifen was initiated. The patient succumbed to an unrelated malignancy after a total of 44 months of treatment.

Despite emerging reports about the potential benefit of hormonal therapy, selective estrogen and progesterone receptor modulators and aromatase inhibitors, for uterine sarcoma, there is a paucity of information regarding the application of these therapies to sarcomas arising at other sites. Our patient survived significantly longer than expected with metastatic retroperitoneal sarcoma. In part this may be due to the survival benefit associated with HR positive tumors, but it may also indicate a role for hormonal therapy which has yet to be explored.

## Introduction

Soft tissue sarcoma (STS) is a broadly defined category that accounts for less than 1% of all reported malignancies (Aragon-Ching & Maki 2012; Collins & Thomas 2011). This group includes high-grade undifferentiated pleomorphic sarcoma, liposarcoma, synovial sarcoma, and leiomyosarcoma (LMS). Amongst these neoplastic processes, LMS accounts for 10% to 24% of all reported STS’s (Aragon-Ching & Maki 2012; Collins & Thomas 2011). Usually, LMS originates in the retroperitoneum, the uterus, and areas surrounding the inferior vena cava, with symptoms attributable to the physical consequence of the sizable tumor mass(Aragon-Ching & Maki 2012). Compared with other sites of origin, LMS originating in the retroperitoneum is associated with a poor prognosis(Aragon-Ching & Maki 2012; Lee et al. 2003).

The therapeutic approach to locally advanced or metastatic STS that is surgically unresectable is combination chemotherapy. However, the expression of various hormone receptors on neoplastic cells provides a potential target for adjuvant and/or alternative treatment modalities. Although hormonal therapy has been recently reported in the treatment of uterine LMS, there is no literature to date on the use of hormonal therapy in LMS that arises in the retroperitoneum. We here report the case of a patient with metastatic retroperitoneal LMS who was treated with chemotherapy followed by hormonal therapy with control of disease for more than 3 years when she died from consequences of unrelated malignancy.

## Case report

A female in the age group 55-60 with a past medical history of schizophrenia and seizure disorder was brought to the hospital after experiencing a fall. She denied fever, weight loss or night sweats. She had a past surgical history of hysterectomy 20 years ago which was performed for fibroids. Her family history was significant for a parent with lung cancer. Her social and personal history were unremarkable. Physical exam revealed a palpable mass in the left axilla. Laboratory tests were within normal limits.

**Figure 1:**
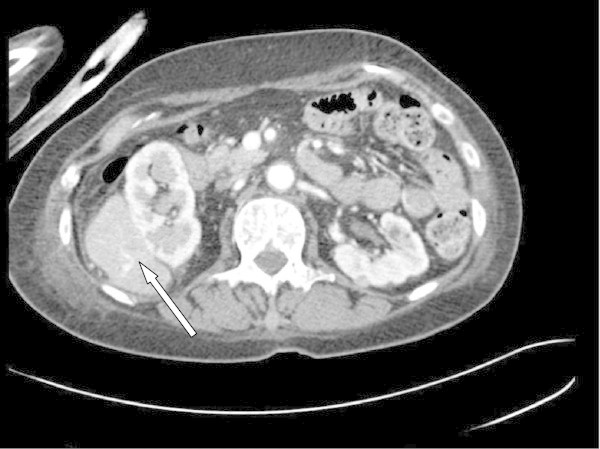
**Initial CT showing retroperitoneal mass compressing on the right kidney.**

**Figure 2:**
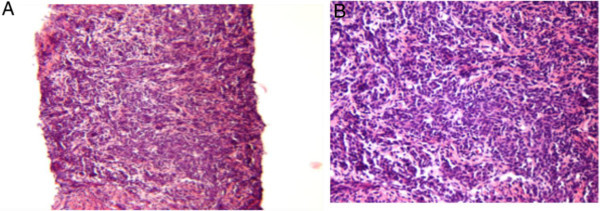
**Histology of the retroperitoneal mass. (A)** Atypical nuclei (10x magnification). **(B)** Spindle cells with atypical nuclei (100x magnification).

**Figure 3:**
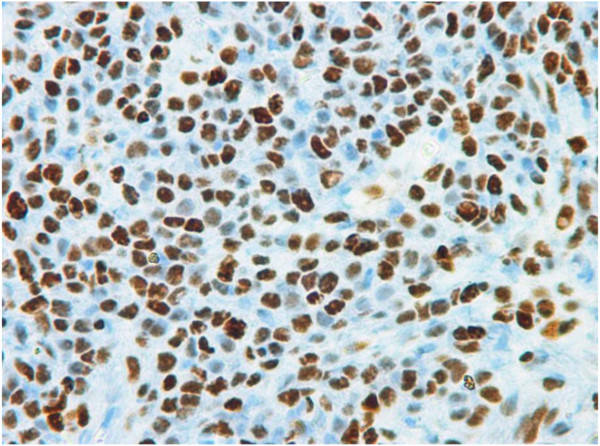
**Positive staining for estrogen receptor.**

CT trauma protocol of the abdomen and pelvis revealed an unanticipated 5×3 cm mass adjacent to the right kidney. Contrast MRI revealed a 5.8×3.7 cm enhancing mass inseparable from the kidney. Its serpentine venous drainage to the IVC magnified the suspicion of malignancy (Figure 1). The CT of the thorax showed a 3.3×2.3 cm node in the left axilla and a 0.9 cm right lung upper lobe nodule. An ultrasound-guided biopsy of the right renal and left axillary lesions was performed which on histopathologic analysis showed atypical nuclei with spindle shaped cells (Figure 2). Immunohistochemistry confirmed leiomyosarcoma with postive immunostaining for smooth muscle actin, muscle actin, vimentin, BCL-2 and CD-99. The lesion was also positive for estrogen and progesterone receptors (Figure 3).

We initiated chemotherapy with Gemcitabine and Paclitaxel. She received a total of 4 cycles and at 29 months her disease remained stable. Tamoxifen was added at that time because of the presence of estrogen receptor in the original biopsy specimen. Repeat CT scan 3 months later revealed a new 5 × 3 cm paraspinal mass. CT guided biopsy identified metastatic malignant melanoma. A skin biopsy on the left arm also showed melanoma. The spine melanoma was managed with palliative radiation therapy and Dacarbazine. The patient expired one year later as a result of complications of melanoma – unrelated to LMS.

## Discussion

The treatment of localized STS includes a combination of surgical resection and adjunctive radiotherapy – an approach that has consistently shown to increase the survival rate. Although promising, this treatment modality is not applicable in cases where patients have advanced metastatic disease. In such cases combination chemotherapy is the indicated treatment (Clark et al. 2005). This approach, however, is burdened by a limited number of effective medications available to clinicians and by poor survival outcomes.

For decades, the combination of Doxorubicin and Ifosfamide has been the mainstay of chemotherapeutic management of metastatic STS. In many patients this regimen is either not applicable due to renal and/or cardiac pathology or due to numerous side effects (Leu et al. 2004). Investigations of alternative regimens have led to the current use of Gemcitabine, in combination with Docetaxel, which has offered favorable outcomes. Studies report an overall response rate for metastatic STS that ranges from 16 to 53%, with a median overall survival that ranges from 12.1 to 17.9 months (Maki 2007). These improved response rates and survival outcomes with Gemcitabine and Docetaxel varies with the histological subtype of the sarcoma. Bay et al., demonstrated that the overall response rate in patients with advanced LMS is 24%, whereas neoplasms of other subtypes show a response rate of only 10% (Bay et al. 2006).

In this case, we presented a patient who was treated with a combination of Gemcitabine and Paclitaxel, following diagnosis of metastatic LMS. Our use of these two compounds resulted in a far better survival outcome than that which has been reported in the literature. We suspect that positive hormonal receptor expression by cells of the LMS may have contributed to the outcome we observed. The association, which has been recognized in cases of gynecologic cancers might also extend to LMS of a retroperitoneal origin. Incidence rates of LMS have been reported to mimic the age specific incidence rate of breast cancer providing further evidence of a role for sex hormones in LMS carcinogenesis (Mastrangelo et al. 2012). Studies of uterine LMS demonstrate that 21–87% and 20–80% are ER and PR receptor positive, respectively (Carvalho et al. 2009). In the case of extrauterine LMS, ER/PR is also expressed with gender defining prevalence. In women, 86% of LMS are ER positive and 86% PR positive. However, in men 22% and 33% of tumors are ER and PR positive, respectively. The expression of steroid hormonal receptors has been found to correlate with a better clinical outcome in both uterine and extrauterine sarcoma (Carvalho et al. 2009; Ioffe et al. 2009). One retrospective cohort study that included 97 patients in various stages of uterine sarcoma who received different treatment modalities, including surgery, chemotherapy and radiation therapy, indicated that patients with ER-positive uterine sarcoma have a median overall survival of 36 months, while ER negative demonstrated a median overall survival of 16 months (Ioffe et al. 2009). Carvalho et al., in their cluster analysis, similarly, found that ER expression correlated with much better survival in cases of both uterine and nonuterine LMS (Carvalho et al. 2009).

The presence of these receptors and the association between ER positivity and better clinical outcome has led to the investigation of hormonal therapy, particularly in cases of uterine sarcoma. There is also in vitro evidence that also supports this approach. For example, in Rhabdomyosarcoma, estrogen receptor beta activation mediates cell proliferation; this effect is blocked by the presence of 4’-OH Tamoxifen (Greenberg et al. 2008). Several authors have focused on the treatment of uterine LMS with Selective Estrogen Receptor Modulators (SERM), selective progesterone receptor modulators (SPRM), and/or with aromatase inhibitors. The aromatase inhibitor, anastrozole, was employed in one case as an adjuvant treatment of metastatic uterine LMS. This approach achieved an objective response of tumor regression for more than one year (Hardman et al. 2007). Although these studies show benefit with hormonal therapy, there is conflicting data. One retrospective cohort study demonstrated a low objective reponse of 9% in advanced uterine LMS that were treated with aromatase inhibitors (O'Cearbhaill et al. 2010).

Because our patient had a hysterectomy 20 years ago, it is clear that the tumor did not originate from uterus*.* To our knowledge, our report is the first to document the application of hormonal therapy in the setting of retroperitoneal LMS. Initiation of tamoxifen was temporally, and perhaps causally, associated with a minimal progression of this extrauterine LMS. However, our observation period was shortened because of the emergence of a second malignancy. Metastatic malignant melanoma was identified three months after the patient was treated with tamoxifen. During the 15 months she was treated with tamoxifen, there was no evidence of LMS progression. The course of her LMS remained distinct from the expected poor prognosis.
